# NPCs in video games: a reflective resource for sports coaches and participant engagement

**DOI:** 10.3389/fspor.2024.1403829

**Published:** 2024-06-14

**Authors:** Chenxi Yin

**Affiliations:** Institute for Sport, Physical Education and Health Sciences (ISPEHS), Moray House School of Education and Sport, University of Edinburgh, Edinburgh, United Kingdom

**Keywords:** coaches, participant engagement, reflection tool, interdisciplinary, training, youth sports

## Abstract

This perspective article explores the potential of non-player characters (NPCs) in video games as a reflective tool for coaches to enhance participant engagement in sports. While coaches traditionally focus on movement skill instruction, their role extends to fostering young people's immersion in sports contexts and potentially contribute to the possibility of lifelong participation. However, challenges persist in translating coaching theory in coach education programs into practice, including the awareness of roles and how to make young people immersion in sports. Integrating elements from video games, where NPCs play pivotal roles in shaping player experiences, presents a possible avenue for re-thinking the role of coach, especially in participation. By drawing parallels between NPCs and coaches, this article advocates for a new reflection tool for coaching roles. Future research should investigate the effectiveness of leveraging NPCs to enhance athlete engagement and motivation, ultimately creating dynamic and inclusive coaching environments that cater to the evolving needs of participants.

## 1 Introduction

The coach plays a crucial role in youth sports as a participation prompter by influencing young athletes' motivation, sense of self, and confidence. Research underscores that coaching transcends mere instruction of sports skills, extending to the cultivation of positive relationships with athletes, which profoundly shapes their sporting experiences (1–4). Effective coaches are adept motivators who foster camaraderie, teamwork, and a shared sense of purpose among young athletes (1, 5–7). Employing positive reinforcement and encouragement, coaches bolster athletes' self-esteem, confidence, and enjoyment of sporting activities (8, 9). These positive experiences for young people could increase the possibility of continuous participation. Conversely, detrimental coaching behaviours, such as fostering unhealthy competition or subjecting athletes to verbal and emotional abuse, can inflict enduring harm on athletes' mental well-being, self-worth, and body image (8, 10). These negative effects contribute to burnout and dropout, even reject participating in sports participation in the rest of their lifelong (11).

Despite the longstanding emphasis in formal coach education coaches should build a positive engagement context for young people, challenges persist in translating this emphasis into practice. One prevalent issue lies in the disproportionate focus on theory learning at the expense of behavioral skills in coach training programs (12–14). For example, “Ideal coaching” should have encompassed a spectrum of competencies, including technical proficiency, interpersonal acumen, leadership qualities, and an understanding of psychological factors influencing athlete performance (12, 15, 16). However, this comprehensive requirement highlights a broader challenge within the coaching practice, as it assumes coaches have access to a variety of resources—such as formal education, mentorship opportunities, and practical experience (13, 17)—that support the development of these diverse skills. Unfortunately, the fragmented nature of these resources means that while some coaches might benefit from certain aspects of this support system, they often lack a cohesive framework that integrates these resources effectively (12, 18), particularly for those new to the profession (17, 19).

Furthermore, a significant number of youth sports coaches lack formal coaching education, relying instead on personal experience to guide athlete development (12, 20). The prevailing trend among coaches prefer informal learning, wherein coaches draw upon their past experiences, engage in self-reflection, and seek insights from peers to augment their coaching knowledge and skills (12, 20–23). It means some valuable theories in formal coach education seem to have not been influenced to some coaches, who have been working in youth sports for a long time. This formal education deficiency, compounded by the competitive youth sports culture, leaves coaches ill-equipped to mentor young athletes effectively (24–26).

Additionally, the current coaching landscape often prioritizes availability over expertise (27). In fact, formal coach education or certification is not a pre-requirement for people being coaches in some countries (28). This means solely depending on developing formal coach education to achieve improving coaches' behavior in youth sports is a challenging and protracted endeavor (12, 29).

Therefore, coach researchers should explore effective ways to influence coaches by considering more diverse and engaging learning resources. These resources could include interactive digital media such as popular video games (30), which have the potential to both attract coaches and effectively connect with young people's participation strategies. Such innovative approaches may serve as viable alternatives or complements to traditional coach education, providing practical and accessible options for coach training in contexts where formal qualifications are less emphasized.

Sports participation, once a cornerstone of leisure activity, now fails with video games, be treated as boring activities, particularly among the Z Generation (31, 32). While the goal of sports participation remains centered on providing positive experiences (33, 34), video games seem to effortlessly captivate young people and adults through immersive gameplay experience (35, 36). To bridge this gap, integration of video game elements into sports is essential (30, 37). A perspective here is: Coaches can adopt a role akin to non-Player Characters (NPCs), providing personalized guidance and motivation to participants in sports participation context. NPCs are integral to the architecture of video games, populating the virtual environment and interacting with players in ways that significantly enhance the immersive experience (38). Thus, by engaging in playing video games and reflecting on the role of NPCs in video games, coaches might gain valuable insights into organizing and enhancing positive sports participation experiences for young people.

## 2 NPCs in video games

Video game settings are pivotal in crafting immersive experiences for players, with meticulous design aimed at enhancing gameplay (36, 39, 40). Central to these settings are NPCs, integral elements that populate the virtual world and interact with players (38, 41).

NPCs are entities within video games controlled by artificial intelligence, distinct from the player's control, and serve multifaceted roles within the game world (42). These roles span from providing information, offering quests, acting as adversaries or allies, to contributing to the overarching narrative of the game (42). By simulating social interactions, NPCs imbue the game world with realism and complexity, creating an environment ripe for exploration (43).

The inclusion of NPCs in video games serves diverse functions, ranging from guiding players through the storyline to enhancing immersion and world-building (44). NPCs facilitate dynamic encounters, furnish context, and furnish background information about the game world, enriching players' understanding and engagement (42, 44).

In essence, NPCs play a crucial role in augmenting the player's experience by infusing depth, interaction, and narrative elements into the virtual environment. Their presence fosters immersion, contextualizes gameplay, and contributes to the dynamic and captivating nature of the gaming experience.

### 2.1 The character of NPCs in video games

NPCs in video games exhibit a diverse array of characteristics and functionalities, each contributing uniquely to the player's experience and immersion within the game world (42). The original (42) identified the common character of NPCs, which are summarized in Table 1.

**Table 1:** Characters of NPCs in video games.

Character	Description
Buy, sell and make stuff, provide services	Across these games, NPCs fulfil the roles of facilitating transactions and providing services, often overlapping, and offering players the opportunity to repair or enhance items.
Provide combat challenges and provide loot	NPCs engage in combat by initiating attacks against either the player or their companions, utilizing a range of weaponry. NPCs that present combat challenges commonly function as standard adversaries and are frequently defeated to acquire loot. Conversely, NPCs offering loot may relinquish items they possess or employ.
Assist the player	NPCs have two main functions: engaging in combat alongside the player or offering guidance and advice. In combat scenarios, NPCs autonomously assist the player. In non-combat situations, they provide assistance, directions, advice, and resources to the player autonomously.
Supply background information (history, lore, cultural attitudes)	NPCs supply background information, contributing to enriching the player's understanding of the game's narrative.
Guard places	Guarding behavior manifests in two primary forms: either NPCs protect a location or individual, hindering player access, or they defend the player or their territories against adversaries.
Dispense quests (or clues of other NPCs’ quests)	These NPCs primarily appear when assignments are issued. The aim is to “give or advance quests”, often forming chains rather than singular objectives.
Make the place look busy	NPCs typically have limited interaction capabilities, although certain specialized NPCs may contribute to creating a more vibrant game world. Their sole function is to enhance the atmosphere, providing no additional services beyond making the world appear bustling.

### 2.2 Theoretical comparison in NPCs and coaches

In this article, I mainly focus on the four characters of NPCs, including “Buy, sell and make stuff/provide services”, “Provide combat/mechanical challenge”, “Assist the player” and “Supply background information”.

#### 2.2.1 Buy, sell, and make stuff and provide services—transactional and supportive roles

When comparing the function of NPCs in video games with the role of coaches in sports, it becomes apparent that both play crucial roles in facilitating player/athlete progression and engagement. NPCs frequently offer players opportunities to repair, upgrade, or acquire items, as well as purchase bonuses to enhance their gaming experience (42). Similarly, coaches in sports act as resource managers, guiding athletes in their training and decision-making processes (7). However, a notable distinction emerges in the level of autonomy granted to players and athletes within these contexts.

In video games, players typically enjoy autonomy in making choices regarding resource acquisition and utilization. Whether it involves repairing and upgrading items or procuring bonuses, players have the freedom to shape their gaming experience according to their preferences and objectives. This autonomy fosters a sense of ownership and empowerment, contributing to deeper immersion and investment in the game world (45).

Conversely, the traditional role of coaches in sports often entails a more directive approach. Some coaches make decisions on behalf of their athletes, serving as the sole arbiters of strategy and resource allocation (46, 47). However, conceptualizing coaches as mere “resource managers” akin to NPCs challenges this conventional paradigm, suggesting a shift towards a more collaborative and player-centered coaching philosophy (48). By relinquishing some control and empowering athletes to make their own choices, coaches can encourage athletes to take ownership of their development and decision-making processes (49, 50).

In redefining the coach's role akin to an NPC, coaches should cultivate a more dynamic and inclusive coaching environment. Rather than dictating every aspect of training and competition, coaches should empower athletes to actively participate in decision-making processes, including goal setting, selecting training methods, and strategizing during gameplay. Embracing this open-minded approach to coaching can not only enhance athlete engagement and motivation but also foster personal growth and development beyond the confines of the sports arena (51).

#### 2.2.2 Provide combat/mechanical challenge—challenge and assistance

Comparing the role of NPCs in video games to that of coaches in sports reveals intriguing parallels in their functions as providers of challenge and encouragement. In video games, NPCs often introduce combat or mechanical challenges, offering players an opportunity to test their skills and advance in the game (42). These challenges are meticulously designed to strike a delicate balance: they must be sufficiently challenging to engage players and foster growth, yet not overwhelmingly difficult to discourage or frustrate them (42, 52). Maintaining this balance is crucial to sustaining player motivation, preventing boredom, and ensuring continuous immersion and engagement in the gaming experience. Similarly, sports coaches also play a pivotal role in orchestrating challenges for athletes, whether through competitive matches, skill development exercises, or strategic drills (50). Recognizing the nuanced balance inherent in the challenges provided by both NPCs in video games and coaches in sports can enlighten coaches on how to foster optimal engagement and growth among their athletes.

#### 2.2.3 Assist the player—facilitative guidance

Similar to NPCs in video games, coaches are responsible for serving as integral facilitators in the “gaming” process, guiding participants through challenges and opportunities for growth. NPCs act as supporting characters, enhancing the immersive experience of the game and providing “just right” assistance for the player to complete the challenge (42). In the context of youth sports, It means when young people face a challenge, a coach needs to provide “just right” assistance for them, rather than replace them to complete the challenge. The assistance from the coach should just keep young people enjoying participating in youth sports without burnout rather than to achieve a challenge (4).

Understanding their role as facilitators rather than participants, coaches should prioritize process over outcomes. Just as NPCs contribute to player immersion and skill development (42), coaches should prioritize helping players hone their abilities and prepare for future challenges in their lives (4). The emphasis lies on creating a supportive and nurturing environment where participants can learn, grow, and thrive, regardless of the outcome of competitions (53).

#### 2.2.4 Supply background information—cultural and narrative contributions

When examining the role of NPCs in video games alongside that of coaches in sports, parallels emerge in their role in conveying narrative knowledge and cultural understanding. In video games, NPCs often serve as repositories of historical background, lore, and cultural perspectives, offering players valuable insights into the game world and its inhabitants (42). Similarly, coaches have the potential to become resource managers of sports culture, imparting not only technical skills but also a deeper comprehension of the kinesio-cultural aspects (54).

However, a concerning trend in current sports participation places a disproportionate emphasis on skill development and basic movement learning, neglecting the significance of understanding the cultural context of the sport (55). While some coaches and athletes may already possess this contextual knowledge or employ strategies integrating cultural understanding into their coaching practices (54), a gap persists within the broader coaching community. Therefore, through critical reflection on the role of NPCs, coaches can potentially glean insights into more effective methods of imparting “kinesio-culture” within the realm of sports.

### 2.3 Summary

Therefore, coaches, much like NPCs, sometimes support athletes by providing guidance, support and resources (56, 57), akin to how friendly NPCs offer help, information, and quests in video games. Conversely, by adopting strategies similar to those of adversarial NPCs—who present challenges and obstacles—coaches also need help athletes develop resilience and strategic thinking (58). This dual role of NPCs, as both allies and challengers, mirrors the multifaceted role of coaches who aim to mentor, guide, and test their young people. Thus, “the NPCs” as informal reflection resources not only provide early-stage coaches with a comprehensive understanding of their role, rather than fragmenting knowledge, but also assist professional coaches in reflecting on what constitutes an engaging participation context for the younger generation. These resources help coaches integrate their practical knowledge to better serve young people.

## 3 Discussion

The comparison between non-player characters (NPCs) in video games and the role of coaches in sports offers promising avenues for both future research and practical applications in coaching. NPCs serve as a compelling reflective tool for coaches, providing valuable insights into effective engagement strategies for athlete participation (42). By adopting an NPC lens, coaches can engage in self-study opportunities aimed at enhancing coaching practices and promoting athlete engagement.

For example, encouraging coaches to engage with video games such as “The Legend of Zelda” allows them to reflect on NPC roles and explore their applicability in sports coaching. The “Legend of Zelda” series is celebrated for its complex world-building and the pivotal roles NPCs play in navigating players through intricate game spaces and narratives. These NPCs not only deliver essential information and pose challenges but also support players in a manner that is engaging and empowering.

This approach is designed to be flexibility, addressing significant obstacles identified in current coaching education, such as the lack of time efficiency and flexibility highlighted by Dawson (59) and Gurgis, Kerr, and Stirling (60). These barriers often deter early career, part-time, and volunteer coaches from engaging fully in traditional training programs. By introducing strategies that facilitate quick and adaptable learning, this method helps coaches understand their roles as facilitators in a context, encouraging young people to autonomously develop their capabilities and strategies.

Furthermore, the evolving landscape of youth sports training necessitates a re-evaluation of participation strategies to align with contemporary lifestyles (61). With modern trends such as video games exerting significant influence, coaches and researchers alike must adapt their methods to resonate with the preferences and interests of young people (30). This adaptation entails exploring the integration of video game elements into sports coaching environments to increase engagement and foster positive outcomes among young athletes.

Moving forward, future research should prioritize investigating the effectiveness of integrating video game elements into sports coaching practices. Such research endeavors can shed light on the potential benefits of leveraging video game elements to enhance engagement, motivation, and effectiveness in coach education. By embracing innovation and drawing insights from diverse sources, coaches can create dynamic and inclusive coaching environments that cater to the evolving needs and interests of the next generation of youth athletes.

## Data Availability

The original contributions presented in the study are included in the article/Supplementary Material, further inquiries can be directed to the corresponding author.
